# Nanoparticle-Based Treatment Approaches for Skin Cancer: A Systematic Review

**DOI:** 10.3390/curroncol30080516

**Published:** 2023-07-25

**Authors:** Michael Joseph Diaz, Nicole Natarelli, Shaliz Aflatooni, Sarah J. Aleman, Sphurti Neelam, Jasmine Thuy Tran, Kamil Taneja, Brandon Lucke-Wold, Mahtab Forouzandeh

**Affiliations:** 1 College of Medicine, University of Florida, Gainesville, FL 32610, USA; 2 Morsani College of Medicine, University of South Florida, Tampa, FL 33602, USA; 3 School of Medicine, Louisiana State University, New Orleans, LA 70112, USA; 4 School of Medicine, Indiana University, Indianapolis, IN 46202, USA; 5 Renaissance School of Medicine, Stony Brook University, Stony Brook, NY 11794, USA; 6 Department of Neurosurgery, University of Florida, Gainesville, FL 32608, USA; 7 Department of Dermatology, University of Florida, Gainesville, FL 32606, USA

**Keywords:** nanoparticle, skin cancer, drug carriers, systematic review, organic, inorganic

## Abstract

Nanoparticles have shown marked promise as both antineoplastic agents and drug carriers. Despite strides made in immunomodulation, low success rates and toxicity remain limitations within the clinical oncology setting. In the present review, we assess advances in drug delivery nanoparticles, for systemic and topical use, in skin cancer treatment. A systematic review of controlled trials, meta-analyses, and Cochrane review articles was conducted. Eligibility criteria included: (1) a primary focus on nanoparticle utility for skin cancer; (2) available metrics on prevention and treatment outcomes; (3) detailed subject population; (4) English language; (5) archived as full-text journal articles. A total of 43 articles were selected for review. Qualitative analysis revealed that nanoscale systems demonstrate significant antineoplastic and anti-metastasis properties: increased drug bioavailability, reduced toxicity, enhanced permeability and retention effect, as well as tumor growth inhibition, among others. Nanoformulations for skin cancers have largely lagged behind those tested in other cancers–several of which have commercialized formulae. However, emerging evidence has indicated a powerful role for these carriers in targeting primary and metastatic skin cancers.

## 1. Introduction

The American Cancer Society estimates that north of 95 thousand new melanomas will be diagnosed in 2023, with an expected death toll of nearly 8 thousand [1]. Risk factors for skin cancer development include positive family history, sun and ultraviolet radiation exposure, genodermatoses, and light complexion, among others. Beyond complications and outcomes, skin cancer also imposes significant financial strains: the annual cost of treating skin cancer has been estimated at over USD 8 billion since 2007, compared to total treatment costs of USD 3.6 billion from 2002 to 2006 [2,3]. Per-case treatment costs for basal cell carcinoma and squamous cell carcinomas diagnosed in 2011, secondary to occupational solar radiation exposure, were greater than CAD 5.5 thousand and 10.5 thousand, respectively [4]. Worse yet, accumulating evidence indicates that primary melanoma survivors are at an elevated risk of developing keratinocyte carcinoma, thereby disproportioning these outcomes across the population [5,6].

**Figure 1:**
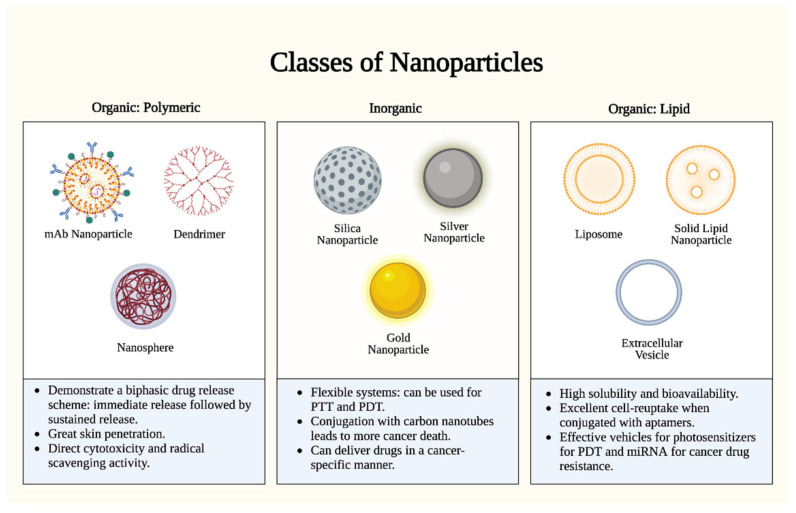
Nanoparticle types. There are two (2) major classes of nanoparticles: organic (polymeric and lipid-based) and inorganic. Each class has specific advantages and mechanisms. Polymeric nanoparticles proffer enhanced bioavailability and a controlled release profile, but they are limited by complex manufacturing and potential toxicity. Inorganic nanoparticles proffer uniquely tunable sizes, shapes, and conjugations, but they are limited by biodegradability concerns and long-term toxicity. Lipid nanoparticles proffer high biocompatibility and biodegradability, but they are limited by reduced payload capacities and stability challenges. PTT: photothermal therapy; PDT: photodynamic therapy. Figure created with Biorender.com.

Although most skin cancers are comfortably excised as localized diseases, therapeutic approaches for locally advanced and metastatic skin cancers are frequently complicated by dysimmune toxicities and limited efficacies [7]. Nanoparticles (NPs)—defined as particles with one dimension < 100 nm—have recently emerged as promising drug delivery systems for such antineoplastic drugs, owing to their enhanced targeting, permeability, and retention [8,9]. NPs have further shown great promise in overcoming multidrug resistance and cytotoxicity barriers intrinsic to current targeted treatment modalities [10,11], with considerable variance attributed to their classification (Figure 1).

However, despite their well-recognized utility in the treatment of aggressive cancers, a comprehensive review of their role and critical potential in the treatment of advanced cutaneous carcinomas remains needed. Here, we appraise and critique the current body of relevant research, with an emphasis on key NP types and their associated benefits, for the development of cutaneous carcinoma therapeutics.

## 2. Methods

### 2.1. Study Design

A systematic review of controlled trials, randomized controlled trials (RCTs), meta-analyses, and Cochrane Review articles was conducted in accordance with the latest Preferred Reporting Items for Systematic Reviews and Meta-Analyses (PRIMSA) guidelines [12]. This review was registered in the international prospective register of systematic review (PROSPERO) (CRD42023442468).

### 2.2. Search Strategy

The Cochrane Library, PubMed, EMBASE, and Scopus databases were broadly queried on 10 March 2023 to retrieve all relevant articles since 1 January 2000. The main keyword search terms were “nanoparticle” and “skin cancer”. Query requirements were restricted to the Title and Abstract fields (“[tiab]”). Search records were managed with Covidence, a web-based collaboration software platform [13].

### 2.3. Eligibility Criteria

All initial search results were subjected to the following inclusion criteria: (1) has a primary focus on nanoparticle usage for primary skin cancer; (2) includes evidence-supported metrics that report prevention and treatment data; (3) details the subject population (i.e., number of human subjects or cell line type). Criteria for exclusion were (1) non-English articles, (2) Abstract-only text (or otherwise unavailable full-text), and (3) yet-published clinical trials. Authors NN, SA, and SJA conducted the eligible article selection process. Disputes were resolved by MJD or JTT.

## 3. Results

### 3.1. Literature Search Results

**Figure 2:**
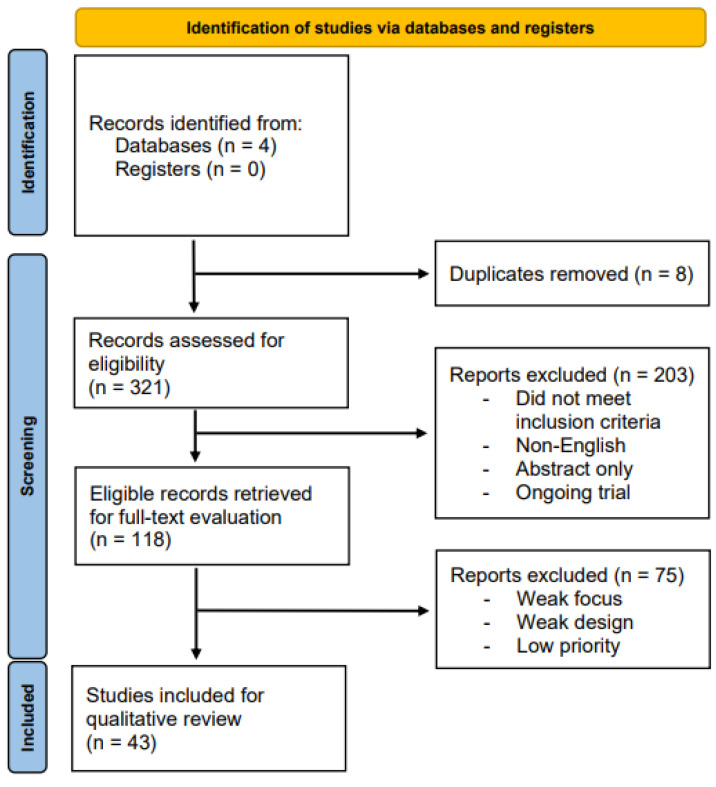
PRISMA flow diagram.

Our initial sensitivity search yielded 329 records, which were preliminarily screened on the Title and Abstract fields. Of these, 211 records were excluded either because of duplication (*n* = 8) or because they failed to meet the stated eligibility criteria (*n* = 203). A total of 118 articles were retrieved for full-text evaluation; 43 articles were selected for review. Figure 2 provides a detailed overview of the search and filtration process.

### 3.2. Primer on Nanoparticle Utility for Skin Cancer Treatment

**Figure 3:**
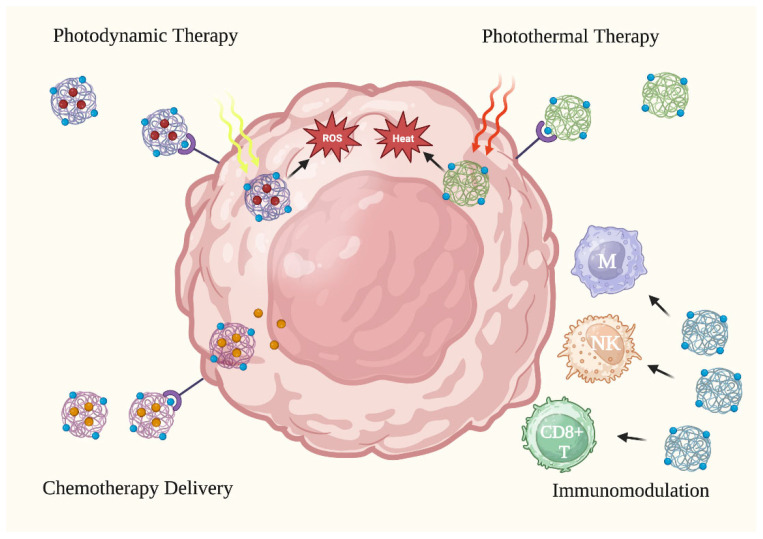
Anti-cancer applications of nanoparticles. Nanoparticles have been tested in skin cancer treatment via four (4) primary treatment methods: photodynamic therapy, photothermal therapy, activating the immune system to attack cancer cells, and improving the delivery of chemotherapy to cancer cells. CD8+ T: CD8+ T cell. M: macrophage. NK: natural killer cell. ROS: reactive oxygen species. Figure created with Biorender.com.

The use of NPs in anti-skin cancer therapy has gained popularity due to their unique physicochemical properties that increase the efficacy of cancer treatments. NPs have shown promising results in skin cancer therapy through various mechanisms of action, such as encapsulating therapeutic moieties in photodynamic therapy (PDT), conducting heat-induced damage in photothermal therapy (PTT), inducing activation and phenotype alteration in immunomodulation, and enhancing drug delivery and penetration in chemotherapy [14,15,16,17]. Various treatment types are illustrated in Figure 3.

Several studies have demonstrated the efficacy of NPs in improving photodynamic therapy. PDT is a non-invasive technique that is used in dermatology, predominantly, for the treatment of actinic keratoses and non-melanoma skin cancers. More recently, their use in cutaneous melanomas has been described. PDT requires three components—the photosensitizer, which must penetrate the skin, excitation light, and oxygen [17]. The photosensitizer is placed inside the targeted cancer cell or in the tumor tissue. When the light is administered, it excites and activates the photosensitizer and generates reactive oxygen species (ROS), which can lead to the apoptosis of the cancer cells and the destruction of tumor tissues [17]. At the same time, however, PDT lacks selectivity, and the ROS can damage surrounding healthy tissues as well [17]. Another issue with PDT is the low bioavailability and delivery of naturally occurring photosensitizers, such as protoporphyrin IX (PphIX). A nanoparticle-based delivery system can encapsulate PphIX into NPs that penetrate the epidermal barrier and allow for targeted delivery to tumor cells [18]. To increase the specificity of nanoparticle delivery, the properties of NPs can be altered to increase interactions with cancer cells. Cancer cells often overexpress integrin receptors (αvβ3) on their cell surface [18]. Targeted delivery of TiO_2_ NPs, which have shown cytotoxicity against melanoma cells, conjugated to a Arg-Gly-Asp (RGD) motif, would thus make PDT more selective, promoting binding with integrin αvβ3 [18,19]. TiO_2_ NPs, conjugated to RGD, exhibit a cytotoxic effect in αvβ3 integrin-expressing mice melanoma cells but not in the normal cells lacking this integrin [18]. In addition to TiO_2_-based PDT, ultra-small hollow silica nanocarriers (HSdots) (~10 nm) can serve as nanocarriers for the targeted topical delivery of photosensitizer zinc phthalocyanine (ZnPc) [20]. ZnPC is a porphyrin that is excited by near-infrared light. When ZnPC-loaded HSdots are conjugated to folic acid, they selectively target squamous cell carcinoma (SCC) regions due to the high number of folic acid receptors in SCC tissues [20]. Photosensitizers can also be loaded into solid lipid nanocarriers for more effective drug delivery and increased selectivity in tumor cells [17]. Phthalocyanine aluminum chloride (AlPc), another example of a photosensitizer, absorbs light between 660 and 770 nm. Mello et al. reported that AlPc, alone, did not permeate the skin, but when it was encapsulated by butter-based solid lipid NPs (SLN-AlPc), there was a permeation of approximately 100% with 8 h of contact [17]. The increase in penetration of the photosensitizer, using the nano-based delivery system, can be attributed to the small size (~17 nm) of SLN-AlPc and the interactions of the solid lipid NPs with the stratum corneum [17]. Once delivered to the tumor site, PDT with SLN-AlPc treatment yielded ROS generation, increased the expression of caspase-3, and decreased the expression of Bcl-2 [17]. Thus, the small size of NPs allows for effective penetration of the epidermal barrier and targeted delivery to tumor cells, leading to selective tumor cell toxicity [17,18].

Photothermal therapy is similar to PDT but without the need for ROS to interact with target cells or tissues. PTT requires the conversion of near-infrared light into heat that can damage cancer cells. PTT can lead to the risk of recurrence or metastasis, however, due to the incomplete elimination of tumor cells [21]. The introduction of NPs into the tumor sites can allow for more efficient destruction of cancer cells through the excitation of NPs, inducing a moderate temperature increase and inducing irreversible cell damage to cancerous cells while minimizing harm to non-target tissues [21]. Magnetite (Fe_3_O_4_) NPs are one such type of NP that demonstrates high absorptivity at near-infrared wavelengths. When Fe_3_O_4_ NPs are activated with near-infrared irradiation, they efficiently convert light into heat and induce apoptosis [21]. NPs can also be utilized in combination therapy involving PTT and immunotherapy. When both types of skin cancer therapies are used together, it can stimulate further tumor shrinkage and reduce the risk of recurrence and metastasis [22]. This was demonstrated in a study that synthesized polydopamine-coated Al_2_O_3_ NPs and injected the NPs directly into B16F10 melanoma allografts in mice for PTT. Then, CpG, a potent stimulator of Th1-type cells, was injected into the mice, so tumor volumes and the number of living mice were recorded. The Al_2_O_3_ within the NP worked with the CpG to trigger a robust cell-mediated immune response that allowed for increased elimination of residual tumor cells. After the combined treatment, 50% of the mice successfully achieved the goal of tumor eradication and survived for 120 days [22]. Gold and silver nanoparticle-assisted PTT, or plasmonic photothermal therapy (PPTT) represents another route. When gold and silver NPs are irradiated, electrons are excited; then, they relax and emit strong localized heat that can destroy nearby surrounding cancer cells [23]. Gold and silver NPs can be combined with carbon nanotubes, which have high thermal conductivity after laser excitation, as effective agents for PPTT [23].

NPs can suppress tumor growth through targeted immunomodulation. Studies have demonstrated NPs altering macrophage polarization towards an M1-like phenotype and increasing CH8+ T cell density [24,25]. Among them, one study utilized nanosized membrane vesicles, known as extracellular vesicles (EVs), which were isolated and purified from the ginseng root, known for their anticancer properties [26]. Mice with B16F10 melanoma were treated with ginseng-derived NPs (GDNPs) therapy [26]. GDNP treatment significantly suppressed melanoma growth in tumor-bearing mice by increasing the presence of M1 macrophages detected in tumor tissue [26]. A separate study found that chitosan-poly(acrylic acid) NPs (CS-PAA), loaded with R848 and MnCl_2_ (R-M@CS-PAA NPs), can also exert an anti-tumor effect by promoting the M1 phenotype [24]. R848 is a toll-like receptor (TLR)7/8 agonist that is known to effectively drive the M1 polarization of tumor-associated macrophages [24]. Administration of R848 alone, however, can cause adverse side effects. Mn^2+^ can also enhance the activation of CD8+ T cells and natural killer cells [24]. R-M@CS-PAA NPs enhanced the polarization of macrophages into the M1 phenotype, and they inhibited the proliferation of B16F10 cells [24]. Another study utilized the immunogenic NPs formulated in micron-sized crystals [25]. Cucumber mosaic virus-like particles, containing tetanus toxin peptide (CuMVTT) NPs covered in a microcrystalline tyrosine (MCT) adjuvant, were injected into tumor sites. CD8+ T cell density was increased in the B16F10 melanoma tumors treated with CuMVTT + MCT [25]. Further, a study sought to enhance immune checkpoint inhibition therapy through antigen delivery by using an E2 protein nanoparticle conjugated to a CpG adjuvant and an MHC-I restricted glycoprotein 100 epitope (gp100). It was found that immunization with CpG-gp-E2 NPs significantly increased CD8+ T cell percentage at the tumor site. The group that received the combination treatment showed a striking increase in survival compared to groups receiving CpG-gp-E2 alone (*p* < 0.001) or anti-PD1 alone (*p* < 0.001) [27]. The surface characteristics of the nanoparticle can also be adjusted to significantly affect the cell entry and intracellular behaviors of NPs to enhance immunomodulation. A new, highly specific inhibitor JQ-1 was shown to be effective in the internalization and reduction in expression of PD-L1 in cancer cells, dendritic cells, and tumor-associated macrophages. A silica core, with etched polydopamine NPs loaded with JQ-1, allows for a sustained release pattern of the drug, reducing the expression of PD-L1 on cancer cells and, simultaneously, activating the immune system, as well as reducing the risk of tumor recurrence and metastasis [16]. The increased roughness of NPs exhibited elevated cellular uptake, allowing the effective entry of JQ-1 into the residual tumor cells. Further, the use of NPs coated with sucrose can prevent aggregation and promote favorable interaction with the tumor microenvironment [28]. When silver NPs were coated with sucrose (S-AgNPs), stability was increased in an aqueous solution, making them suitable intravenous agents. S-AgNPs also enhanced the antitumor activity of anti-PD-1 treatment and significantly increased tumor-infiltrating CD8+ T cells [28].

Moreover, organic NPs have been evidenced to deliver chemotherapy drugs, in a targeted way, to prevent toxicity to healthy cells, enhance drug penetration depth, and provide targeted drug delivery [14,15]. Fe_2_O_3_ NPs can be conjugated to L-cysteine (L-cys) to increase stability, and then, they can be bound to doxorubicin (Dox). Binding Dox to L-cys-coated Fe_2_O_3_ NPs allowed for efficient Dox delivery after internalization into melanoma cells. After the rapid uptake of Fe_3_O_4_-L-Cys-Dox NPs in melanoma cells, within 3 h of treatment, there were noticeable apoptotic effects detectable at 48 h post-exposure [14]. Another study demonstrated the preparation of chitosan NPs to enhance the tumor penetration capability of 10-hydroxycamptothecin (HCPT) [15]. Chitosan is a cationic polysaccharide that can interact with negatively charged biological membranes by electrostatic interaction [15]. Thus, when HCPT is encapsulated into the core of chitosan-coated NPs, the charge interaction with biomembranes allows for penetration deep into the tumor and promotes internalization by tumor cells [15]. Further, in vitro analysis displayed sustained release patterns, whereas HCPT, alone, exhibited a very rapid release rate [15].

Table 1 provides a comprehensive summary of 15 full-text articles selected for describing the general utility of NPs for the treatment of skin cancers.

### 3.3. Inorganic Nanoparticles

Inorganic nanoparticles, such as titanium dioxide [18], zinc oxide [21], carbon nanotubes, gold nanoparticles, silver nanoparticles, and silica nanoparticles have been extensively tested as therapeutic drug delivery systems for skin cancer prevention (i.e., sun protection) and treatment.

#### 3.3.1. Gold Nanoparticles (AuNPs)

AuNPs have been shown to penetrate and accumulate effectively in tumoral tissue due to their high biocompatibility, customizable surface properties, and their ability to be conjugated to other molecules [29,30]. AuNPs effectively absorb photon energy following laser exposure and convert it to heat, which can dissipate and evoke damage to nearby cancer cells, making them effective to utilize in photothermal therapy (PTT) [30]. PTT experiments using AuNPs have consistently shown prolonged survival in melanoma tumor models, as well as effective tumor regression due to the cell death of skin cancer cells, with limited damage to surrounding healthy tissue [30,31,32,33]. AuNPs have also proven efficacious in stabilizing photosensitizers in photodynamic therapy (PDT) and providing enhanced cellular uptake, leading to increased amounts of skin cancer cell apoptosis and singlet oxygen generation [34]. The function of AuNPs has been enhanced by coating them with other materials or conjugating them to other molecules [29,32,33,35,36]. Coating AuNPs with materials, such as red blood cell membranes, has allowed for a significant reduction in the rapid physiological clearance of NPs by the monocyte–macrophage system [35]. The conjugation to cell-penetrating peptides, such as tumor-targeting adaptor folic acid, has allowed for enhanced cellular uptake and elevated PTT effects [32]; conjugation to cell-targeting molecules, such as anti-HER2 and melanoma-associated antigen antibodies, allows for selective killing and uptake via melanoma cells [33,36]; conjugation to other antitumor therapies, such as betulin, has resulted in increased growth inhibition and the proliferation of melanoma cells in vitro [29]. Utilizing AuNPs with other anti-skin cancer therapies has proven efficacious due to the properties that allow them to selectively enter into tumor cells and inhibit the growth of cancer cells. Further exploration of the use of AuNPs with varying therapies may continue to prove beneficial.

#### 3.3.2. Silver Nanoparticles (AgNPs)

AgNPs exhibit high biocompatibility, resistance to oxidation, and a wide array of antimicrobial and anti-inflammatory activities [37,38]. AgNPs, when compared to AuNPs, have been found to have a greater photodynamic effect in PDT and generate more cytotoxic reactive oxygen species following irradiation, resulting in higher extinction coefficients in tumor cells, higher ratios of scattering to extinction, and higher field enhancement [37]. AgNPs, similarly to AuNPs, exhibit good optical absorbance and low toxicity towards normal cells, so they are a viable material for use in PTT as well [23,39]. AgNPs used alone in PTT, against a murine model of melanoma, have been shown to invoke up to 45% necrosis of tumor cells, and when conjugated to carbon nanotubes, they can invoke up to 70% necrosis [23]. AgNPs coated with bovine serum albumin have also been utilized in PTT, and they are able to invoke nearly complete tumor cell death at temperatures above 45 °C while also proving to have inhibitory effects on the angiogenesis of tumor cells [39]. AgNPs have also shown greater anti-tumor effects upon optimization with other materials or when synthesized in different manners. A study has shown that AgNPs synthesized from *Fusarium incarnatum* fungal extracts have an ability to inhibit tyrosinase activity (the main enzyme in the biosynthesis of melanin), in melanoma cells, in a dose-dependent manner [38]. In addition to maximizing their cytotoxic effects, it is equally as important to maximize the ability of AgNPs to reach cancer cells. This has been done by coating AgNPs with materials that make them more likely to be taken up into cancer cells, such as polyvinylpyrrolidone (PVP). PVP-AgNPs have been shown to decrease the genotoxic effects of AgNP therapy, as well as allow for an enhancement in the rates of cancer cell apoptosis [40]. New and improved methods of enhancing the use of AgNPs with other cancer therapies, new methods of synthesis, or ways to coat the molecules are being reported in the literature, and they may lead to the development of new and improved methods of treating human skin cancer.

#### 3.3.3. Silica Nanoparticles (SiNPs)

SiNPs are silica core polyethylene glycol shell NPs with the ability to function as drug delivery molecules and circumvent the dose-limiting toxicities posed by many anti-skin cancer therapies. They are cleared by the kidneys and have low tissue uptake in most organs, making them an efficacious adjunct therapy in the treatment of skin cancer [41]. SiNPs exhibit favorable pharmacokinetics and low tissue accumulation, so they have been optimized with cell-targeting molecules to directly target cancer cells [41,42]. There is a method that has been shown to be efficacious is conjugating SiNPs to melanocortin-1 receptor, targeting alpha melanocyte-stimulating hormones. This method was found to exhibit effective tumor penetration and distribution in vivo, as well as accumulation and retention of SiNPs in melanoma tumors in vivo [42]. Another method, utilizing the same alpha melanocyte-stimulating hormone functionalization, has shown enhanced efficacy of targeted radiotherapy in melanoma models via efficient internalization of the NPs, as well as favorable tumor uptake and retention. Melanoma-bearing mice treated with this therapy were found to exhibit higher lengths of survival compared to control groups [41]. SiNPs have also been utilized as a system of drug delivery via loading the NPs with anti-cancer drugs, such as verteporfin, cisplatin, or resveratrol [43,44,45]. SiNPs loaded with verteporfin were found to nearly abolish the appearance of lung micrometastases, and they showed reduced lymphangiogenesis of a murine model of melanoma [43]. SiNPs loaded with cisplatin led to reduced toxicity in healthy cells when compared to cisplatin therapy alone, and they are effective at successfully inhibiting tumor growth in in vitro and in vivo studies [45]. SiNPs loaded with resveratrol led to the increased bioavailability and solubility of resveratrol, leading to efficiency cytotoxicity in the cells of two melanoma cancer lines [44]. The potential drawback of loading SiNPs with other anti-cancer therapies is that resveratrol, in the previous study, was found to crystallize in the pores of the NPs, potentially preventing total release of the drug [44]. Overall, SiNPs provide favorable pharmacological characteristics that may be utilized alongside other therapies to enhance their efficacy and improve outcomes. Further evaluation may provide better insight into how to best exploit their advantageous characteristics.

Table 2 provides a comprehensive summary of 18 full-text articles selected for the review of inorganic NPs in the context of skin cancer treatment.

### 3.4. Organic Nanoparticles

The use of organic nanoparticles, such as liposomes, solid lipid nanoparticles, polymeric nanoparticles, dendrimers, mAb nanoparticles, and extracellular vesicles have also shown unique promise in skin cancer research. Compared to inorganic NPs, the organic NPs report greater functionalization with cancer-targeting ligands and increased drug loading versality. Noteworthy findings from studies on lipid and polymeric NP-based treatment in the context of skin cancer have been summarized below.

#### 3.4.1. Lipid Nanoparticles (LNPs)

LNPs are a drug delivery system composed of ionizable lipids, allowing enhanced solubility and bioavailability while reducing toxicity. The search strategy yielded nine studies evaluating LNP use in the treatment of melanoma. LNPs loaded with temozolomide (TMZ), anti-parasitic benzimidazole, plumbagin, *Zataria multiflora* essential oil, and *Mentha longiflora* and *Mentha pulegium* essential oils have demonstrated cytotoxicity against melanoma cells in vitro. *Mentha longiflora*/*Mentha pulegium*-LNPs and *Zataria multiflora*-LNPs reduced cell viabilities to under 10% and 13%, respectively [46,47]. Benzimidazole-LNPs induced cancer cell apoptosis, generated reactive oxygen species, and inhibited Bcl-2 expression in cancer cells while sparing toxicity among healthy HEK293T cells [48]. In vivo murine studies were additionally conducted with plumbagin-loaded lipid–polymer hybrid NPs (LPNP) and TMZ-LNPs. Intravenous administration of plumbagin-LPNPs resulted in the disappearance of 40% and regression of 10% of B16-F10 melanoma tumors [49]. Similarly, TMZ-LNPs inhibited B16-F10 melanoma growth and vascularization without apparent toxicity [50]. Selective targeting capability of aptamer-associated LNPs has also been evaluated. Compared to free SA and SA-LPNPs lacking CD20 aptamers, CD20+ melanoma cells demonstrated significantly greater uptake of CD20-SA-LPNPs, resulting in enhanced cytotoxicity in vitro and in vivo with murine models [51]. Aluminum-phthalocyanine-LNPs also demonstrated strong intro photodynamic activity among melanoma cells, suggesting utility in photodynamic therapy [17].

In addition to primary cytotoxicity, LNPs have been evaluated as a therapeutic strategy to combat drug resistance, specifically, among BRAF-mutant melanoma cell lines [52,53]. Authors have discovered microRNAs, namely miR-204-5p and miR-199b-5p, involved in drug resistance development [53]. The overexpression of miR-204-5p and miR-199b-5p inhibit melanoma cell growth in vitro, both alone and in combination with BRAF/MEK inhibitors (MAPKi), suggesting their antagonism of resistance. LNPs loaded with microRNAs effectively inhibited their target oncogenes, Bcl-2 and VEGF-A, impaired melanoma cell proliferation and viability, and potentiated the efficacy of MAPKi. In addition, Fattore et al. evaluated the microRNA-LNPs among mouse models injected with A375 (*n* = 7) or M14 (*n* = 10) melanoma cell lines [52]. While the NPs strongly potentiated the effects of target therapies, greater tumor inhibition was observed in A375 mice compared to M14 mice. Authors subsequently assessed miR-204-5p and miR-199b-5p expression and found greater uptake in A375-derived tumors [52]. The results of these two studies demonstrate both the in vitro and in vivo potentiation of targeted melanoma therapy, demonstrating the potential utility of microRNA-LNPs in resistant melanoma. However, LNP uptake and efficacy may be dependent on the tumor-derived cell line.

#### 3.4.2. Polymeric Nanoparticles

Polymeric NPs are composed of biocompatible polymers, which may be synthetic or natural in origin. In addition to the previously described studies conducted by Zeng et al. [51] and Sakpakdeejaroen et al. [49], employing lipid–polymer hybrid NPs, three studies evaluated the therapeutic potential of polymeric nanoparticle (PNP) in the management of melanoma. A 2021 study developed and evaluated an α-mangostin-loaded PNP topical gel formulation [54]. Measurable outcomes included drug release, skin permeation, cytotoxic effects against B16-F10 melanoma cells, and in vitro radical scavenging activity [54]. The PNPs demonstrated biphasic drug release, which is characterized by immediate release followed by sustained release. Confocal microscopy on rat skin demonstrated α-mangostin-PNP penetration up to 230.02 µm compared to dye solution penetration of only 15.21 µm [54]. Compared to free α-mangostin gel, the α-mangostin-PNPs depicted a significantly greater cytotoxic and antioxidant effect (*p* < 0.05) [54]. After 48 h, melanoma cell viability was 18.50% for α-mangostin-PNPs compared to 80.87% for free α-mangostin gel [54]. These findings suggest that α-mangostin-PNPs may be a promising approach for the treatment of skin cancer due to their biphasic drug release, enhanced skin permeation, direct cytotoxicity, and radical scavenging activity.

Similarly, Ferraz and colleagues observed concentration-dependent cell death against B16-F10 melanoma cells with S-nitromercaptosuccinic acid-PNPs [55]. S-nitrosothiols depict therapeutic potential as ^•^NO donors. S-nitromercaptosuccinic acid-PNPs also demonstrated selective toxicity, as melanoma cells were reportedly more sensitive to cell death compared to healthy melanocytes. Cytotoxicity was characterized by caspase-dependent apoptotic features, oxidative stress, mitochondrial superoxide production, and protein thiol group oxidation. In contrast, free S-nitromercaptosuccinic acid and empty chitosan NPs failed to exhibit cytotoxicity [55]. In addition to in vitro analysis, Xiong et al. assessed in vivo efficacy of dacarbazine-PNPs with a murine model [56]. Dacarbazine-PNPs were further modified with nucleic acid aptamer AS1411 for the active targeting of malignant melanoma (dacarbazine-PNPs-Apt). In vivo analysis found the greatest tumor growth inhibition with dacarbazine-PNPs-Apt compared to free dacarbazine and dacarbazine-PNPs, although all three active treatments significantly reduced tumor growth compared with the saline and blank nanoparticle control groups. Specifically, the aptamer allowed for the targeting of melanoma cells, while tumor acidity released dacarbazine [56]. Collectively, these studies demonstrated the ability of PNPs to enhance skin penetration and drug release, as well as exhibit direct cytotoxicity against malignant melanoma cells, both in vitro and in vivo. Furthermore, aptamer modification may be useful to enhance drug localization, thereby increasing drug delivery and efficacy while reducing cytotoxicity to healthy cells. Dendrimers are spherical polymeric macromolecules with highly branched characterizations. Their unique branches allow for the targeting of nucleic acids.

Table 3 provides a comprehensive summary of 12 full-text articles selected for review of organic NPs in the context of skin cancer treatment.

## 4. Discussion

This systematic review aimed to evaluate developments made in NP therapy, specifically, for cutaneous carcinomas. Nanotechnology may improve current skin cancer therapies due to its unique properties as a primer. NP size is advantageous for epidermal barrier penetration, resulting in the selective toxicity of tumor cells [17,18]. NPs can be used to deliver photosensitizers and drugs to targeted tumor cells. Moreover, NP-based delivery systems have the advantage of customization with conjugation and coating, allowing for increased specificity and decreased off-targeting. These alterations improve the outcomes of PDT, PTT, immunotherapy, chemotherapy, and combination therapy, as evident through multiple studies discussed throughout this review. When conjugated with certain motifs, NPs can increase the specificity of PDT by promoting NP-photosensitizer binding with integrins that are overexpressed on cancer cells [18]. Conjugation with other molecules can increase stability of the NP delivery system and prolong the release of chemotherapeutic drugs [14,15]. The coating of certain inorganic NPs, such as AuNPs with red blood cell membranes or AgNPs with bovine serum albumin, can optimize the effects of NP-based delivery [35,57]. These studies showcase the versatility of NPs when modified using conjugation and coating. Such results suggest that NP-based delivery systems can improve the efficacy of skin cancer treatment due to the tailored properties of the NPs.

*Inorganic NPs*. AuNPs, AgNPs, and SiNPs are among some of the inorganic NPs that may prove favorable for drug delivery systems. AuNPs and AgNPs are viable options for PDT and PTT since they emit localized heat to destroy cancer cells and exhibit low toxicity to normal cells [23]. Conjugation and coating only serve to enhance these NP-based delivery systems. With PTT, AgNPs conjugated with carbon nanotubules showed improved tumor destruction when compared to AgNPs only [23]. Unlike AuNPs and AgNPs, SiNPs can be used for drug delivery. Loading SiNPs with anti-cancer drugs, such as cisplatin, led to reduction in normal cell toxicity due to the targeting nature of the NPs [45]. However, SiNPs may also prevent the total release of anti-cancer drugs, such as resveratrol, due to the drug crystalizing within the pores of the NPs [44]. These basic results suggest that SiNPs may be a feasible option for drug-based skin cancer therapy, but further studies are warranted to improve delivery. AuNPs and AgNPs also exhibit promising results, both individually and in conjunction with other therapies. New methods for the conjugation and coating of AuNPs and AgNPs may prove beneficial for skin cancer treatment, and further exploration of may lead to the development of improved PDT and PTT.

*Organic NPs*. LNPs and polymeric NPs are organic NPs that show potential for skin cancer treatment. LNPs have demonstrated cancer cell apoptosis, tumor growth inhibition and regression, and significant cell-reuptake when conjugated with anti-CD20+ aptamer [17,49,50,51]. LNPs may also be used in PDT as aluminum-phthalocyanine-LNPs that efficiently delivered the photosensitizers, resulting in apoptosis of the cancer cells. Based on the successful results of previous studies, continued research in this topic may yield beneficial results for multiple types of therapies. PNPs have demonstrated effective skin penetration and drug release, resulting in the cytotoxicity of skin cancer cells [54,55,56]. PNP–aptamer conjugation may also improve drug delivery to cancer cells and decrease normal cell toxicity. Studies suggest that the effects of α-mangostin-PNPs, such as biphasic drug release, enhanced skin permeation, and direct cytotoxicity, make it a potential approach for PNP-based therapy [54]. Considering the versatility and efficacy of NPs, nanotechnology is highly promising for the future development of skin cancer therapies.

**Figure 4:**
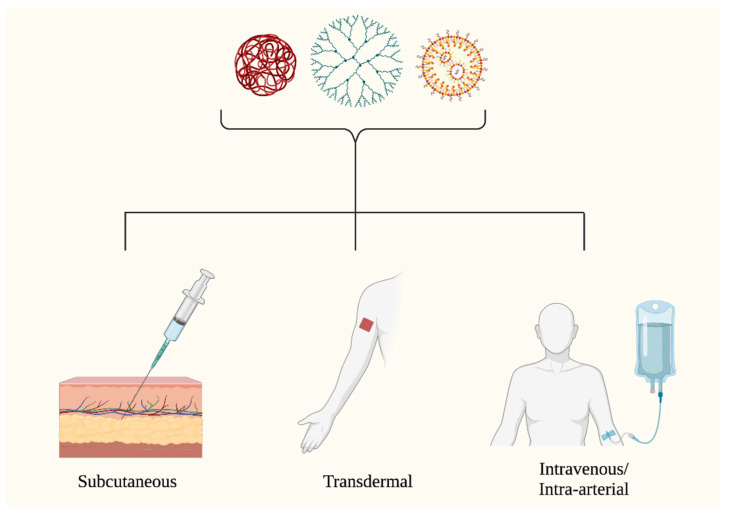
Routes of administration. There are four primary routes of administration for NP-based skin cancer therapy: subcutaneous, transdermal, intravenous, and intra-arterial. Figure created with Biorender.com.

*Delivery.* Nanoparticle drug delivery for skin conditions can be administered via several routes, including subcutaneous, intravenous, and intra-arterial injection (Figure 4); however, the means of drug administration may alter its biodistribution and efficacy [58].

Nanocarriers consisting of lipids, metals, or polymers represent a promising feature of transdermal drug delivery for skin disease therapeutics, as they have been employed to successfully increase drug penetration, prolong drug release, and facilitate targeted drug delivery to specific locations of the skin in vivo [59]. Nanoparticle-based technology offers the potential to expand the use of transdermal routes of administration that minimize the pain and invasiveness associated with the other routes, and it allows for deeper skin penetration [60]. Due to the novelty of this paradigm, few clinical trials have been conducted on skin cancer patients. A recent phase 1/2 clinical trial investigated the safety, efficacy, and tolerability of a topical nanoparticle paclitaxel ointment on breast cancer patients with cutaneous metastases, and it found the treatment to be safe and well tolerated. There is a shortage of approved clinical trials for NP-based drug delivery systems due to the toxicity issues that warrant further research to ensure high safety for the implementation of nanomedicine in the clinical setting. Although NP-based treatments overcome many of the barriers associated with conventional therapy, the systemic side effects, such as nausea and argyria, should be assessed prior to further clinical authorization. With the advancements in nanoparticle-based treatments, new routes of drug administration should continue further exploration to enhance precision therapeutics and optimize drug delivery [61].

## 5. Conclusions

Herein, we conducted a systematic review regarding advancements in nanoparticle utility for advanced cutaneous carcinomas. The introduction of NP-based delivery systems has ushered in a wealth of new insights into the active and passive targeting of cancer cell populations. While their self-therapeutic properties have yet to be fully established, the potential benefits of NP-based therapies are clear. It is likely that we will soon see significant strides made in the development of multifunctional, stimuli-responsive, and mutation-selective NPs. Future investigation should aim to elucidate the mechanism and the predisposing factors of toxic NP aggregation and degradation, as well as the protective in vivo countermeasures. The strengths of this review were its adherence to the PRISMA and Cochrane Handbook for Systematic Reviews of Interventions guidelines, where applicable, and the integration of multiple study designs in the eligibility criteria. Potential limitations include the lack of quality assessment and language restriction.

## Figures and Tables

**Table 1:** Summary of studies retrieved to develop a scoping primer on the utility of nanoparticles to target and treat skin cancer (*n* = 15).

Author, Year	Study Design	Key Findings
Toderascu, 2023 [14]	Controlled trial, mouse (B16F10) and human (A375) metastatic melanoma cells	− L-Cysteine (L-Cys)-coated magnetic iron oxide nanoparticles (NPs) loaded with doxorubicin (Dox) induce a 95–98% apoptosis in B16F10 and A375 melanoma cells
Guo, 2020 [15]	Controlled trial, Mice melanoma B16F10 and B16F1 cells	− 10-Hydroxycamptothecin encapsulated in chitosan nanoparticles significantly enhance tumor penetration and significantly inhibited the progression of tumor (*p* < 0.05)
Xue, 2022 [16]	Controlled trial, Mice with B16F10 melanoma	− Silica-based core–shell nanoparticles (JQ-1@PSNs-R) improved the therapeutic effect of photothermal immunotherapy
Mello, 2022 [17]	Controlled trial, Mice with B16F10 melanoma	− Lipid based nanoparticles associated with aluminum-pthlaocyanin (SLN-AlPC) allowed for the distribution of hydrophobic drugs and thus a potential system to transport photosensitizers − Compared to control, B16F10 cells treated with SLN-AlPC produced a higher amount of reactive oxygen species (*p* < 0.0001)
Dayan, 2018 [18]	Controlled trial, Mice melanoma B16F10 cancer cells	− The TiO_2_–DLDH^RGD^ nanobiocomplex improves TiO_2_—based photodynamic therapy through an integrin targeted delivery approach and controlled-release of ROS − In the presence of TiO_2_–DLDH^RGD^ there was a cytotoxic effect observed in αvβ3 integrin-expressing mice melanoma cells (B16F10) following UVA illumination
Bilkan, 2023 [19]	Controlled trial, Human melanoma cell line	− Titanium oxide nanoparticles combined with UV-A radiation significantly increased the percentage of apoptotic cells (*p* < 0.05)
Dam, 2019 [20]	Controlled trial, Human cutaneous SCC lines	− ZnPC-loaded HS dots led to greater than 90% SCC death after one laser exposure with photodynamic therapy with a 2- to 3-fold increase in caspase 2 expression, indicating apoptosis
Wang, 2022 [21]	Controlled trial, BALB/c mice	− Iron oxide nanoparticle clusters in combination with photothermal therapy significantly inhibited the growth of implanted tumor xenografts in BALB/c mice by reducing tumor volumes by 77.8%
Chen, 2018 [22]	Controlled trial, Mice melanoma B16F10 cancer cells	− After photothermal therapy and immunotherapy treatment with polydopamine-coated Al_2_O_3_ nanoparticle and injection with CpG adjuvant, 50% of mice achieved the goal of tumor eradication and survived for 120 days
Behnam, 2018 [23]	Controlled trial, Mice with B16F10 melanoma	− Ag NPs decorated on carbon nanotubes significantly reduced melanoma tumor size after plasmonic photothermal therapy
Liu, 2021 [24]	Controlled trial, Mice with B16F10 melanoma	− Chitosan-poly(acrylic acid) nanoparticles (CS-PAA NPs) loaded with R848 and MnCl_2_ (R-M@CS-PAA NPs) modulates the immune cells in the tumor microenvironment by promoting the maturation of APCs and significantly increasing the proportion of CD8+ T cells and significantly increased the polarization of M2 macrophages to M1 macrophages
Mohsen, 2022 [25]	Controlled trial, Mice with B16F10 melanoma	− Cucumber mosaic virus-like particles containing a tetanus toxin peptide (CuMVTT) conjugated to a microcrystalline tyrosine adjuvant injected into B16F10 melanoma tumors significantly inhibited tumor growth and significantly increased CD8+ T cell infiltration (*p* < 0.0001)
Cao, 2019 [26]	Controlled trial, Mice with B16F10 melanoma	− Ginseng-derived nanoparticles promoted the polarization of M2 to M1 phenotype and significantly decreased tumor growth
Neek, 2020 [27]	Controlled trial, Mice with B16F10 melanoma	− More than 50% of the mice treated with protein E2 nanoparticles contain CpG oligonucleotide and glycoprotein 100 melanoma antigen epitopes (CpG-gp-E2) in combination with anti-PD-1 treatment were tumor-free
Kuang, 2022 [28]	Controlled trial, mouse (B16F10) and human (A375) metastatic melanoma cells	− Ag nanoparticles coated with sucrose (S-AgNPs) in combination with treatment with PD-1 mAbs showed potent antitumor effects with mild systemic immunotoxicity

**Table 2:** Summary of articles reporting on the utility of inorganic nanoparticles for skin cancer treatment (*n* = 18).

Author, Year	Study Design	Key Findings
Mioc, 2018 [29]	Case-control, Human melanoma cell line A375	− Betulin coated gold nanoparticles presented a dose-dependent cytotoxic effect and induced apoptosis in all tested cell lines
Suarasan, 2022 [30]	Case-control, Agarose-based skin biological phantoms and B16:F10 melanoma cells	− AuNPs in the form of nanotriangles were found to produce the greatest PTT effects
Bonamy, 2023 [31]	Case-control, Human melanoma cell line SK-MEL-28	− AuNPs synthesized by green chemistry are less cytotoxic than gold nanoparticles alone
Zhang, 2018 [32]	Case-control, Murine melanoma cell line B16-BL6	− Necroptosis of melanoma cells using PTT is temperature dependent and is observed in gold nanorod (GNR)-mediated PTT
Li, 2020 [33]	Case-control, B16 mouse melanoma cells	− Gold nanoparticles conjugated to a monoclonal antibody to melanoma-associated antigens targeting melanoma achieved complete eradication of tumors in a murine model of melanoma.
Chi, 2020 [34]	Case-control, A431 cells and HaCat cells	− PDT with gold nanoparticles conjugated to 5-ALA significantly suppressed cell viability, increased cell apoptosis and singlet oxygen generation in both HaCat and A431 cells.
Zhao, 2022 [35]	Case-control, B16-F10 melanoma cells	− Red blood cell membrane-camouflaged gold nanoparticles had an antiproliferation and apoptosis-inducing effect on B16-F10 cells which might be mediated by oxidative stress of reactive oxygen species.
Jeon, 2019 [36]	Case-control, B16-F10 melanoma cells	− Gold nanoparticles conjugated to anti-HER2 antibodies were found to show condensation of nuclei and translocation of apoptosis-inducing factor and cytochrome c from mitochondria into the nucleus and cytoplasm, respectively.
Malindi, 2022 [37]	Systematic review	− Silver nanoparticles have the potential to enhance photodynamic therapy for melanoma treatment.
Himalini, 2022 [38]	Case-control, Human skin melanoma SK-MEL-3 cells	− Mycosynthesized AgNPs can be considered as effective anti-melanogenic agents.
Kim, 2021 [39]	Case-control, B16F10 murine melanoma cells	− Bovine serum albumin (BSA) coated silver nanoparticles showed a considerable light-to-heat conversion ability, suggesting their utility as photothermal agents. − BSA-silver nanoparticles showed marked cytocidal effects on melanoma cells
Behnam, 2018 [23]	Case-control, B16/F10 melanoma cell lines injected into mice	− Integration of carbon nanotubes with silver nanoparticles could enhance the optical absorption of carbon nanotubes and improve tumor destruction in plasmonic photothermal therapy.
Valenzuela-Salas, 2019 [40]	Case-control, B16-F10 murine skin melanoma cells from C57BL/6J mice	− Nanoparticles coated with polyvinylpyrrolidone (PVP) were shown to have antitumor activity with a survival rate almost 4 times higher than treatment with cisplatin alone.
Zhang, 2020 [41]	Case-control, B16F10 murine melanoma cells	− Silica nanoparticles conjugated to alpha-melanocyte stimulating hormone were found to have enhanced treatment efficacy and a clear survival benefit in melanoma models.
Chen, 2018 [42]	Case-control, B16F10 melanoma bearing mice	− Melanocortin-1 receptor targeting silica nanoparticles were found to have favorable in vivo renal clearance kinetics and receptor-mediated tumor cell internalization allowing for therapeutic applications
Clemente, 2021 [43]	Case-control, B16-F10 melanoma bearing mice	− Mesoporous silica nanoparticles were found to half lymphangiogenesis when compared to control, and mesoporous silica nanoparticles loaded with verteporfin were found to nearly abolish lymphangiogenesis.
Marinheiro, 2021 [44]	Case-control, Human A375 and MNT-1 melanoma cell cultures	− Mesoporous silica nanoparticles loaded with resveratrol were found to have a decreased melanoma cell viability in vitro.
Drača, 2021 [45]	Case-control, B16F1 melanoma cell lines and B16F1 melanoma bearing mice	− Mesoporous silica nanoparticles loaded with cisplatin were found to significantly diminish tumor volume in syngeneic model of mouse melanoma induced in C57BL/6 mice.

**Table 3:** Summary of articles reporting on the utility of organic nanoparticles for skin cancer treatment (*n* = 12).

Author, Year	Study Design	Key Findings
Mello, 2022 [17]	In vitro study with murine B16-F10 melanoma cells	− Aluminum-phthalocyanine-LNPs demonstrated strong intro photodynamic activity among B16-F10 melanoma cells − Photoactivated aluminum-phthalocyanine-LNPs exhibited a 50% cytotoxicity concentration of 19.62 nM; treatment induced apoptosis
Kelidari, 2022 [46]	In vitro study	− *Mentha longiflora*/*Mentha pulegium*-LNPs reduced cell viability
Valizadeh, 2021 [47]	In vitro study	− *Zataria multiflora*-LNPs demonstrated a dose-dependent antiproliferative effect
Movahedi, 2021 [48]	In vitro study	− Benzimidazole-LNPs induced cancer cell apoptosis, generated reactive oxygen species, inhibited Bcl-2 cancer cell expression, created morphological change of cancer cells, and significantly reduced migration
Sakpakdeejaroen, 2021 [49]	In vivo murine study (*n* = 25 total, *n* = 5 each group)	− Intravenous administration of plumbagin-LPNPs resulted in the disappearance of 40%, regression of 10%, and stability of 20% B16-F10 melanoma tumors (*n* = 5)
Clemente, 2018 [50]	In vivo study with B16-F10 melanoma in C57/BL6 mice	− TMZ-LNPs inhibited B16-F10 melanoma growth and vascularization, without observed toxicity
Zeng, 2018 [51]	In vivo study and murine in vivo study	− –salinomycin-loaded lipid-polymer nanoparticles with anti-CD20 aptamers displayed effective delivery of salinomycin to CD20+ melanoma cells
Fattore, 2020 [52]	In vivo murine study (*n* = 7 A375; *n* = 10 M14)	− microRNA-loaded LNPs potentiated the effects of target therapies − Greater tumor inhibition with A375 mice compared to M14 mice
Fattore, 2020 [53]	In vitro study	− microRNA-loaded LNPs inhibited Bcl-2 and VEGF-A, impaired melanoma cell proliferation and viability, and potentiated the efficacy of MAPKi
Md, 2021 [54]	In vitro and in vivo rat study	− α-mangostin-loaded polymeric nanoparticle gel enhanced α-mangostin delivery observed by a significant cytotoxic effect and antioxidant effect compared to α-mangostin gel (*p* < 0.05)
Ferraz, 2018 [55]	In vivo murine study	− S-nitromercaptosuccinic acid-PNPs induced cell death against B-16-F10 melanoma cells in a dose-dependent manner − Free S-nitromercaptosuccinic acid and empty chitosan nanoparticles did not exhibit cytotoxicity
Xiong, 2022 [56]	In vitro study	− Dacarbazine-loaded targeted polymeric nanoparticles demonstrated greater A875 melanoma growth inhibition compared to free dacarbazine and dacarbazine-PNPs
